# Skin avulsion injuries caused by the application of adhesive drapes during total knee arthroplasty: A case report

**DOI:** 10.1097/MD.0000000000011049

**Published:** 2018-06-15

**Authors:** Bin Yang, Chuanzhi Xiong, Zhihua Lu, Jingcheng Wang

**Affiliations:** a Yangzhou University; b Department of Orthopedics and Institute of Orthopedics, Clinical Medical College of Yangzhou University, Subei People's Hospital of Jiangsu Province, Yangzhou, China.

**Keywords:** adhesive drapes, degloving injury, surgical-site infection

## Abstract

**Rationale:**

The use of adhesive drapes has decreased the incidence of surgical-site infections.

**Patient concerns:**

Despite the obvious benefits of the proper use of drapes, there remain many potential risks.

**Diagnose:**

A 66-year-old man with a history of left knee osteoarthritis and varus deformity underwent total knee arthroplasty at our hospital, upon removal of the adhesive drape by delicate peeling and warm saline lavage, a large area of the skin avulsion happened.

**Interventions:**

A standardized set of care measures were applied to the wound to avoid wound infection.

**Outcomes:**

After 6 weeks of treatment, the avulsed wound showed no signs of infection and had undergone re-epithelialization.

**Lessons:**

Individuals with high-risk skin avulsion injuries should be provided with preventive measures and the necessity of continuous application of adhesive drapes should be further studied.

## 1 Introduction

Surgical-site infections (SSIs) are associated with serious morbidity, mortality, and increased resource utilization.^[1]^ Nursing measures for elderly patients and improved nutritional status may shorten the duration of preoperative hospital stay and reduce the incidence of infection.^[1,2]^ Other measures including preoperative antiseptic showers, hair removal, skin preparation, and antimicrobial medical device in the operation room have been reported to reduce SSI rates.

At present, the application of adhesive drapes for the purpose of covering the surgical field is required in the majority of arthroplasty procedures. The application of adhesive drapes is generally believed to provide many benefits, particularly in reducing the incidence of SSI.^[3,4]^ Adhesive drapes may also be used to delineate the surgical field. Lastly, wound irrigation may be more conveniently performed as plastic drapes allow for easy fluid run off.^[3]^

We report a case of a degloving injury caused by the use of iodophor-impregnated adhesive drapes during total knee arthroplasty (TKA). A standardized set of care measures were applied to a large area of the wound to avoid wound infection. The patient in the case report was informed and expressly agreed to publish the case report. We had also obtained a written informed consent with the patient's signature. The report was in conformity to the Declaration of Helsinki and approved by The Ethics Committee of Subei People's Hospital.

## 2 Case

A 66-year-old man with a history of left knee osteoarthritis and varus deformity was scheduled to undergo TKA at our hospital. The left lower extremity was prepped using a topical solution, which was to disinfect skin 2 times with chlorhexidine alcohol skin disinfectant (mainly consist of 4.5 g/L chlorhexidine acetate and 70% alcohol). Let the skin air dry, and then place the iodophor-impregnated antimicrobial incise drape on the operation area, which included application of an iodophor-impregnated antimicrobial incise drape. Draping was performed using standard aseptic procedure.

**Figure 1:**
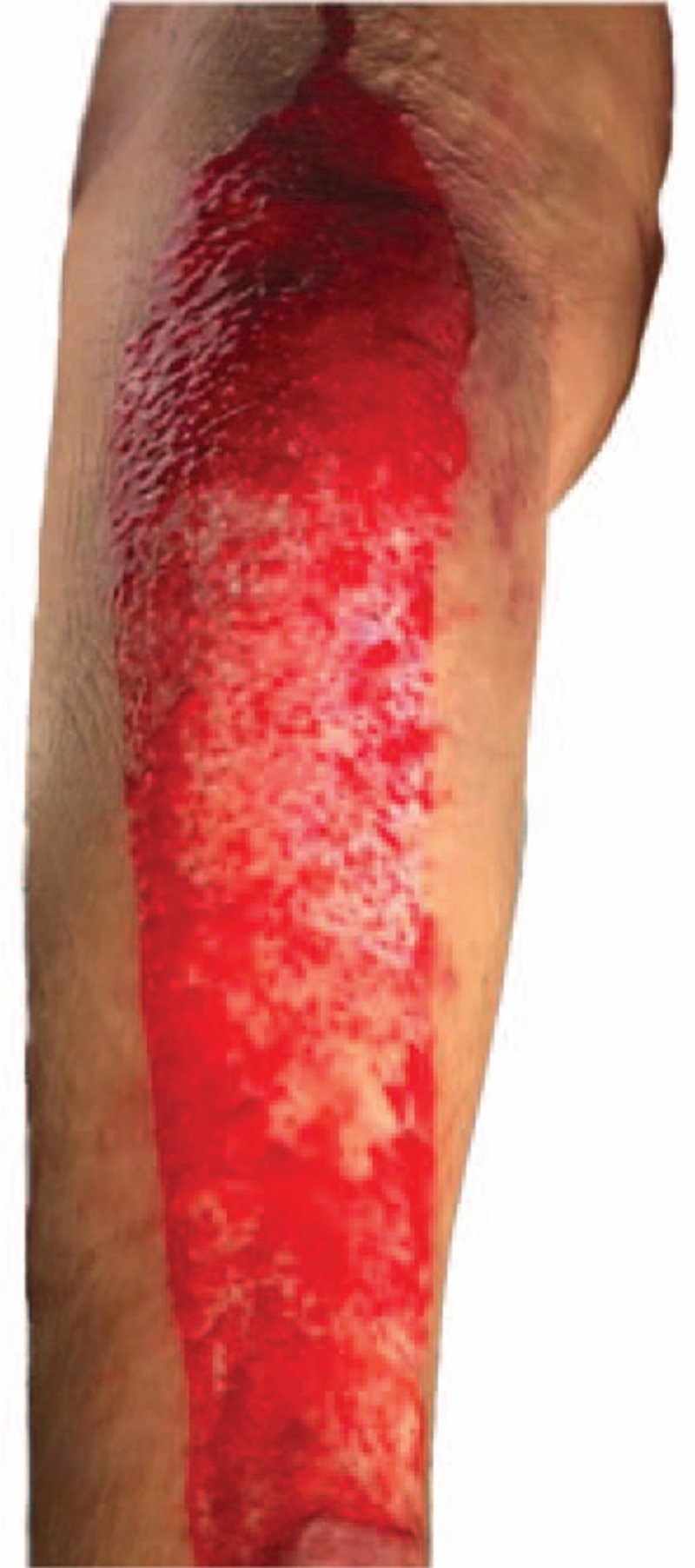
A large area of the skin avulsion with extensive punctate hemorrhage happened.

Subsequently, surgery was performed using conventional surgical procedures. At the end of the operation, the skin incision was sterilized again and sutured. Upon removal of the adhesive drape by delicate peeling and warm saline lavage, a large area of the skin avulsion happened. The extensive punctate hemorrhage was visible (Fig. 1). The skin avulsion was in the posterior part of the patient's left crus. The avulsion area was approximately 6.5 × 30 cm.

**Figure 2:**
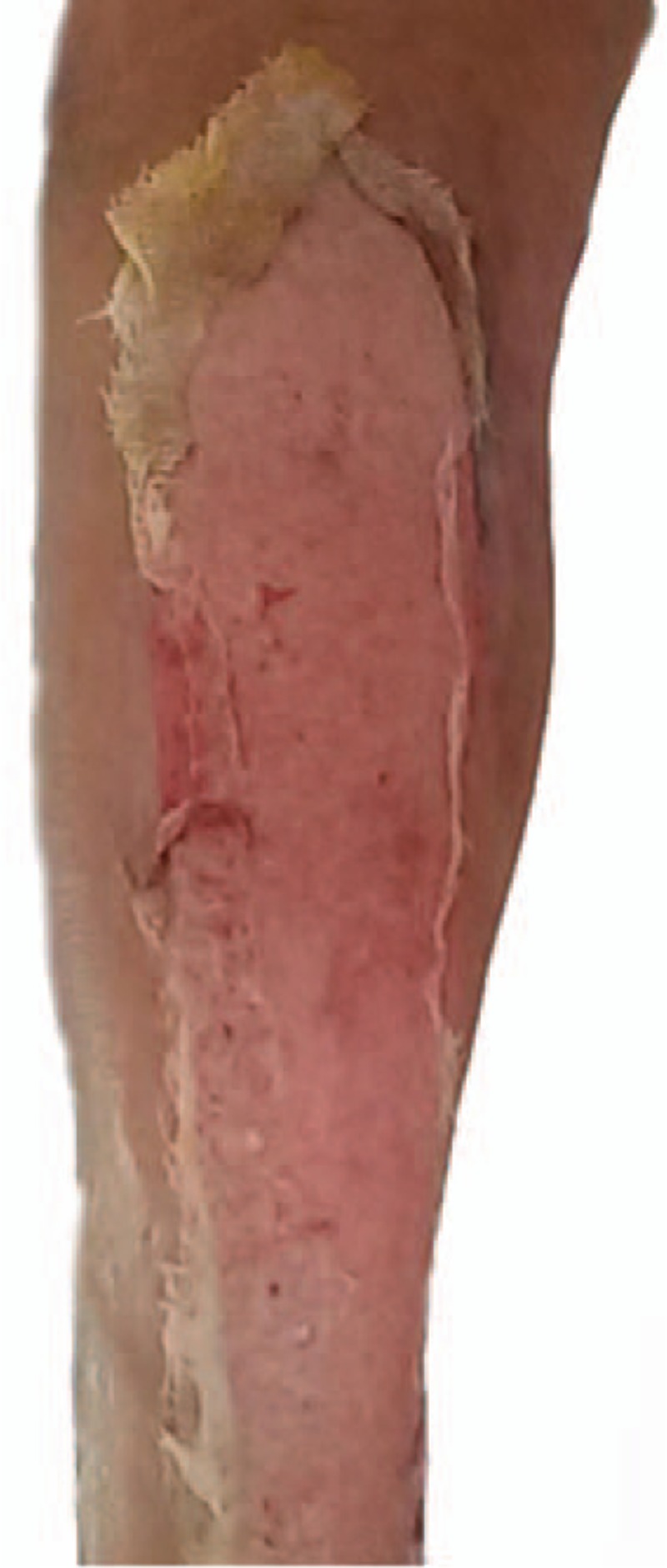
On postoperative day 4, the light yellow liquid exudes and the localized inflammatory response was visible.

**Figure 3:**
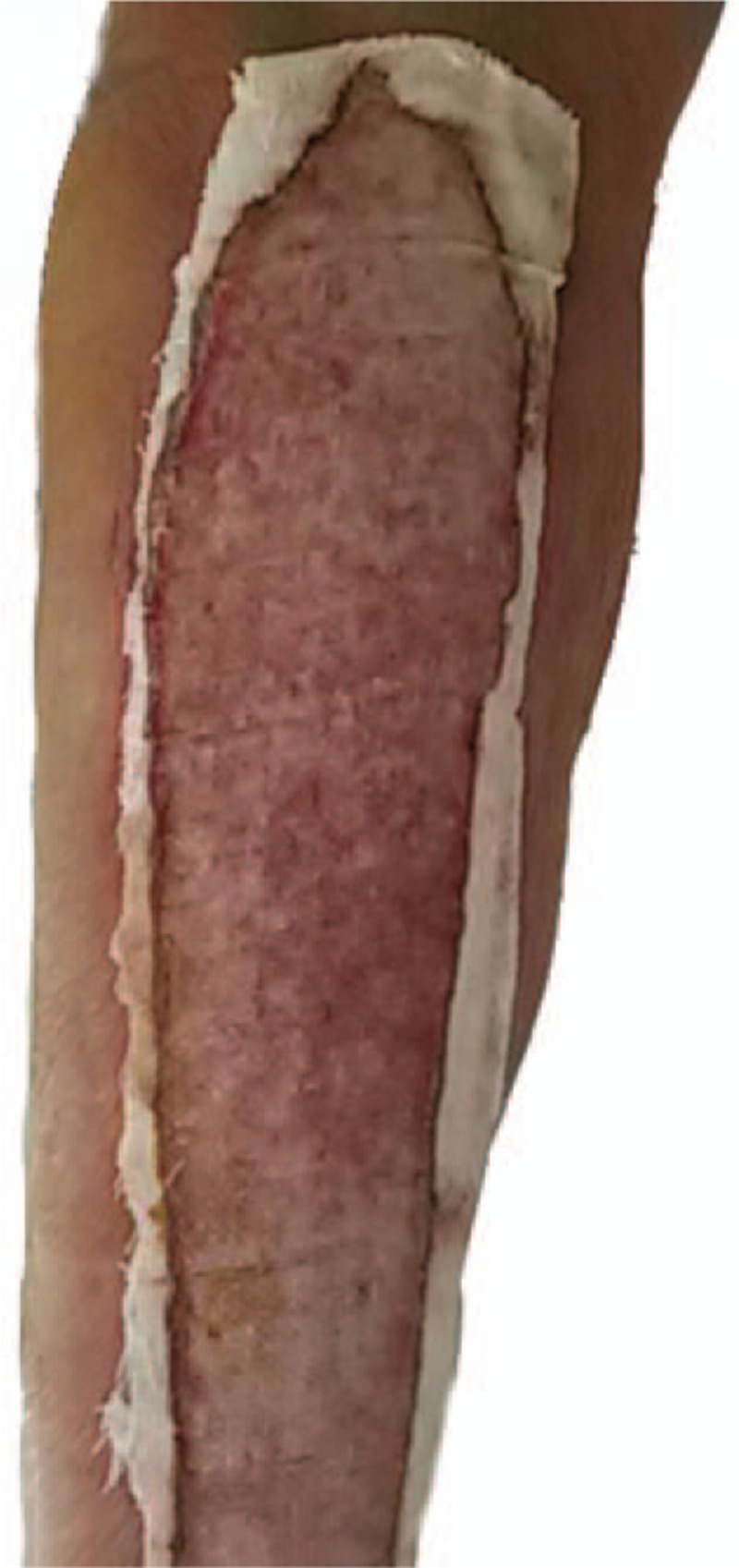
On postoperative day 7, the exudate decreased and the inflammatory response was reduced.

**Figure 4:**
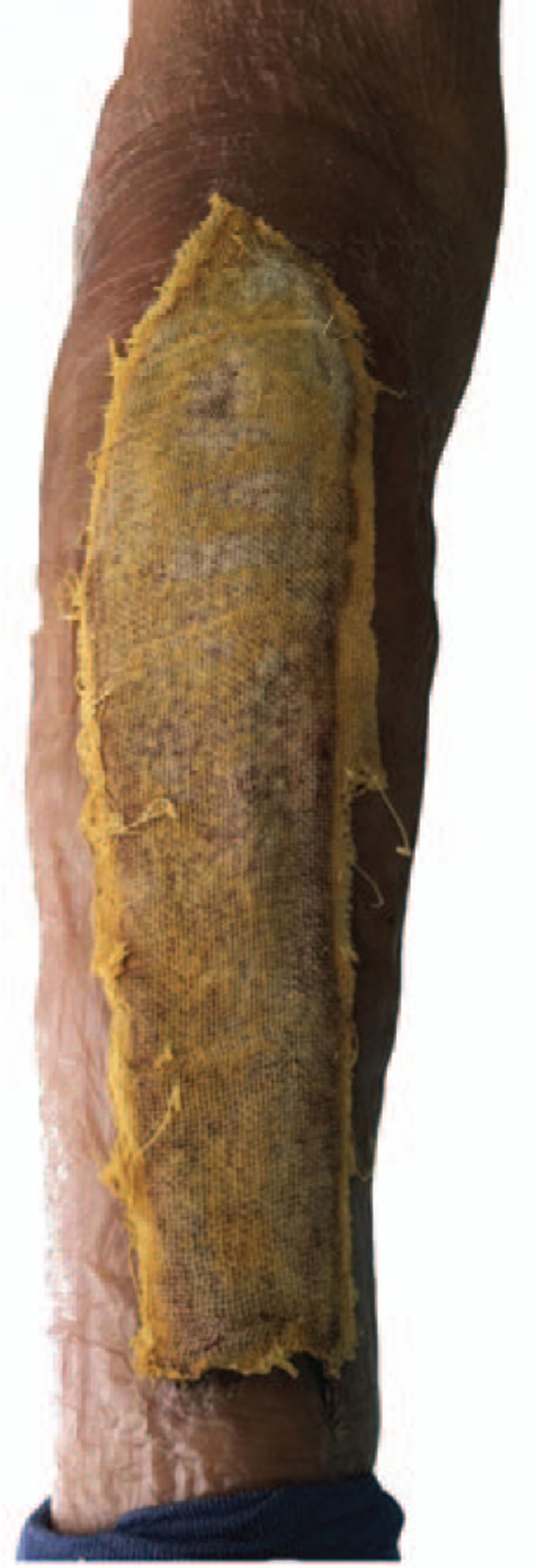
On postoperative week 2, the wound was dry without exudate and obvious inflammatory reaction.

**Figure 5:**
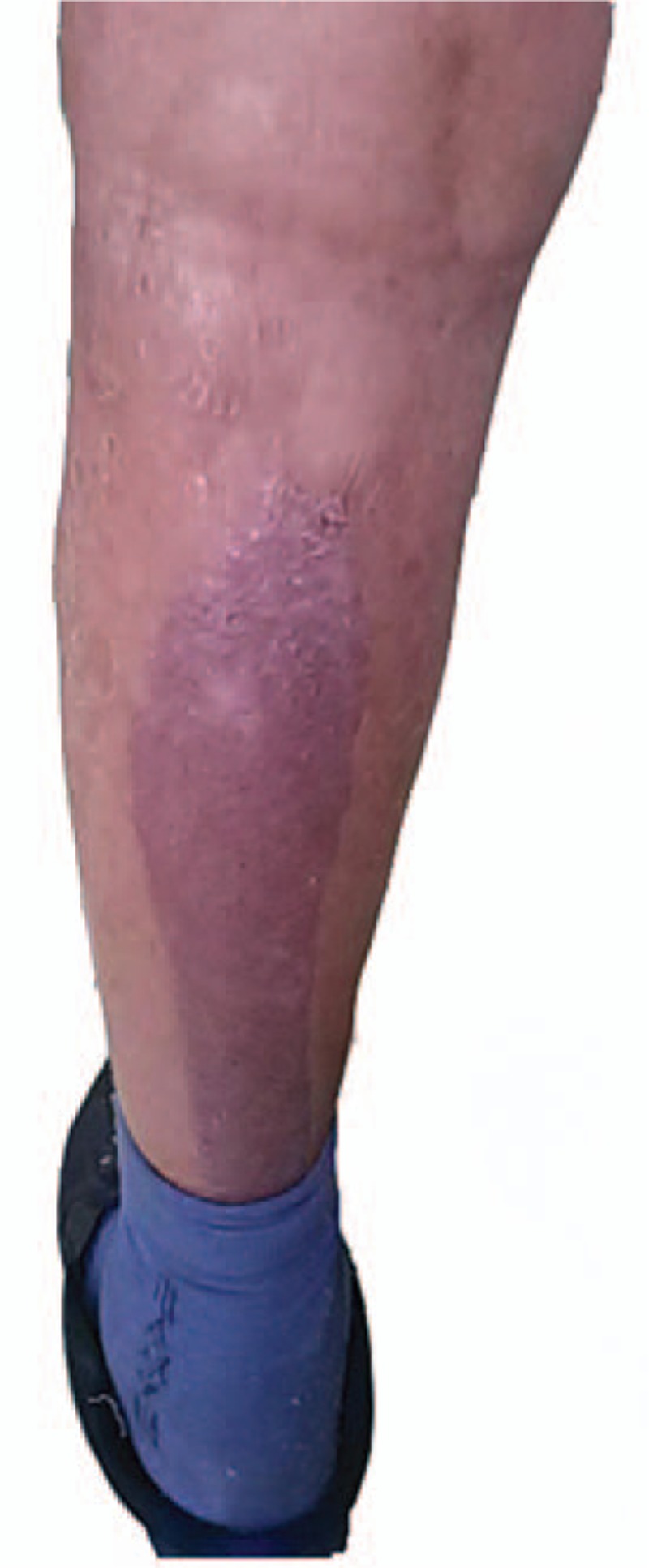
After 6 weeks of treatment, the avulsed wound had undergone re-epithelialization without signs of infection.

Petrolatum gauze was used to dress the areas of epidermal avulsion. Routine postsurgical dressing (gauze, cotton pad, and bandage) was applied to the incision. On postoperative day 2, an initial dressing change was performed. We consulted with doctors from other relevant departments regarding the avulsion injury who advised on the applicable treatment measures. Based on these measures, we asked the patient to raise the affected leg and keep the wound dry. Sulfadiazine cream was applied to the area of epidermal avulsion. Dressing changes were performed daily. On postoperative day 3, oral cefixime therapy of 100 mg bid was initiated as part of the anti-infection treatment. On postoperative day 4, the light yellow liquid exudes and the localized inflammatory response was visible (Fig. 2). On postoperative day 7, the exudate decreased and the inflammatory response was reduced. There was no evidence of infection (Fig. 3). On postoperative week 2, the wound was dry, and there was no exudate and no obvious inflammatory reaction (Fig. 4). The patient was discharged from hospital on postoperative 2 weeks and was required to change the dressing every 3 days until the avulsion area was dry. After 6 weeks of treatment, the avulsed wound showed no signs of infection and had undergone re-epithelialization (Fig. 5).

## 3 Discussion

The SSI is an important concern in clinical practice and typically indicate the failure of surgical procedures. Microbial contamination is possible during any operation. According to epidemiologic studies, the incidence of total joint arthroplasty (TJA) revisions is predicted to increase with the growing number of the TJA procedures likely to be performed in the future.^[5,6]^ According to Nationwide Inpatient Sample data, periprosthetic joint infection has become a significant factor related to TJA revisions. At the same time, its increasing incidence is likely to result in a growing economic burden and a greater burden on hospital and personnel resource utilization compared with other types of revision surgery.^[7–9]^

There are 3 main measures to minimize SSI, which include a series of preoperative, intraoperative, and postoperative measures.^[10,11]^ Some medical comorbidities have been proven to be associated with increased risk of severe infection, predominantly including diabetes, obesity, rheumatoid arthritis, and immunosuppression. Particular measures must be adopted to control these comorbidities before proceeding with TJA to optimize the health of the patient and facilitate operative success.^[12,13]^ Special care should be taken to control intraoperative risk factors, which include operating room traffic, use of antibiotics, and surgical-site preparation.^[10]^ Postoperative factors predominantly include the use of blood transfusions, wound and hematoma drainage, and so on.

The application of adhesive drapes is common during preparation of the surgical site. Previous studies have shown that bacterial penetration, lateral migration, and proliferation are prevented by adhesive drapes.^[3]^ However, its usage to reduce the rate of SSI remains a controversial topic. A recent Cochrane review indicated that there is no evidence that plastic adhesive drapes decrease the incidence of SSI.^[14]^ Common plastic adhesive drapes have been reported to be associated with a risk of skin avulsion injuries.^[15]^ Iodophor is a stimulant chemical agent; when combined with stimulation, impregnation, friction, and pressure, it can make the skin more prone to burns and superficial ulcers.^[16,17]^ Therefore, iodophor-impregnated adhesive drapes are more likely to cause degloving injuries.

Aging can cause structural changes in the skin and consequently lead to impaired skin function. In the present case, the patient was over 65 years of age, an age associated with flattening of the dermal–epidermal junction and other age-related changes in the dermis leading to decreased resistance to shear.^[18]^ In addition to advanced age, other risk factors can lead to skin tears, including diabetes, poor nutrition, and chronic steroid use. In high-risk patients, greater care is essential while removing adhesive drapes. A wet gauze ball, almost the size of a broad bean, held in a vessel clamp, can be used to separate the drapes from the edge of the wound to ensure that the skin is not peeled off while removing the drapes, which is the author's opinion.

## 4 Conclusion

The use of adhesive drapes to reduce the rate of SSI remains controversial. In high-risk patients, the use of adhesion curtains is more likely to result in complications such as skin avulsion injuries, skin burns, and superficial ulcers. High-risk individuals should be provided with a preventive protocol, including the identification of high-risk individuals by the cut-off point of dermis and cautionary removal technique.^[19]^ In light of these various factors, the continued use of adhesive drapes in TJA and other surgeries may warrant further study.

## Author contributions

**Data curation:** Chuanzhi Xiong.

**Methodology:** Zhihua Lu.

**Writing – original draft:** Bin Yang.

**Writing – review & editing:** Jingcheng Wang.
